# Meta-Analysis of Antinuclear Antibodies in the Diagnosis of Antimitochondrial Antibody-Negative Primary Biliary Cholangitis

**DOI:** 10.1155/2019/8959103

**Published:** 2019-06-10

**Authors:** Qian Zhang, Zhiqiang Liu, Shanshan Wu, Weijia Duan, Sha Chen, Xiaojuan Ou, Hong You, Yuanyuan Kong, Jidong Jia

**Affiliations:** ^1^Department of Gastroenterology, Beijing Friendship Hospital, Capital Medical University, Beijing Key Laboratory for Precancerous Lesion of Digestive Disease, National Clinical Research Center for Digestive Diseases, Beijing 100050, China; ^2^The United Innovation of Mengchao Hepatobiliary Technology Key Laboratory of Fujian Province, Mengchao Hepatobiliary Hospital of Fujian Medical University, Fuzhou 350025, China; ^3^Clinical Epidemiology and EBM Unit, Beijing Friendship Hospital, Capital Medical University, National Clinical Research Center for Digestive Diseases, Beijing 100050, China; ^4^Liver Research Center, Beijing Friendship Hospital, Capital Medical University, Beijing Key Laboratory of Translational Medicine on Liver Cirrhosis, National Clinical Research Center for Digestive Disease, Beijing 100050, China

## Abstract

**Objective:**

The diagnostic value of antinuclear antibodies (ANAs) including anti-gp210 and anti-sp100 for primary biliary cholangitis/cirrhosis (PBC) has been widely reported. However, their diagnostic performances for antimitochondrial antibody- (AMA-) negative PBC were less well elucidated. Therefore, the aim of the current meta-analysis was to evaluate the diagnostic accuracy of ANAs in patients with AMA-negative PBC.

**Materials and Methods:**

Literature on the diagnostic value of biomarkers for AMA-negative PBC was systematically searched in PubMed, MEDLINE, EMBASE, and the Cochrane Library. The qualities of the retrieved studies were assessed by the Quality Assessment of Diagnostic Accuracy Studies-version 2 (QUADAS-2) scale. Pooled sensitivity and specificity of the biomarkers were calculated with random-effects models. The areas under the summary receiver operating characteristic (AUSROC) curves were used to evaluate the overall diagnostic performance of ANAs.

**Results:**

A total of 11 studies (400 AMA-negative PBC patients and 6217 controls) were finally included in the meta-analysis. ANAs had an overall sensitivity of 27% (95% CI: 20%, 35%) and specificity of 98% (95% CI: 97%, 99%). The pooled sensitivities for anti-gp210 and anti-sp100 were 23% (95% CI: 13%, 37%) and 25% (95% CI: 13%, 43%), respectively, and their specificities were 99% (95% CI: 97%, 100%) and 97% (95% CI: 93%, 98%), respectively.

**Conclusions:**

ANAs exhibited high specificity but low sensitivity and therefore could be used as reliable biomarkers to reduce the necessity of liver histology.

## 1. Introduction

Primary biliary cholangitis (PBC) (formerly known as primary biliary cirrhosis) is a chronic intrahepatic cholestatic disease which is histologically characterized by progressive nonsuppurative cholangitis [1, 2]. Antimitochondrial antibody (AMA) is a diagnostic hallmark for patients with PBC [3, 4], providing an over 90% sensitivity and specificity. According to major international guidelines, the diagnosis of PBC can be confidently made in patients with clinical, biochemical, and radiological evidence of intrahepatic cholestasis if they are positive for AMA [3, 4]. However, for patients negative for AMA, the diagnosis of PBC has to be based on typical pathological features of this disease [5, 6].

Recently, other serum markers for diagnosis of PBC have been widely investigated [7–9]. Anti-gp210 and anti-sp100 are two biomarkers associated with severe disease and poor outcome [10–12], which require more devoted attention in the diagnosis of PBC. The major glycoprotein, anti-gp210, suggests that it is integrated into nuclear membranes with a small number of polypeptides in the nuclear pore complex [13]. Anti-sp100 is the main antigenic target of multiple nuclear dot (MND) reactivity and consists of a 53 kDa nuclear protein with transcription-stimulating activity [14, 15]. It is justified to evaluate the diagnostic accuracy of ANAs in patients with high suspicion of PBC but negative for AMA, with the aim to reduce the necessity of liver biopsy which is invasive in nature and potentially causes serious complications [16, 17]. A meta-analysis indicated that antinuclear autoantibodies (ANAs) including anti-gp210 and anti-sp100 were found in 30%-50% of patients with PBC [18] but did not specifically address their diagnostic performances for AMA-negative PBC. Another review article did summarize the diagnostic values of ANAs for AMA-specific PBC but did not aggregate the data with meta-analysis [19].

Therefore, we conducted this meta-analysis to evaluate the diagnostic performances of ANAs (with a specific focus on anti-gp210 and anti-sp100) for AMA-negative PBC.

## 2. Materials and Methods

### 2.1. Search Strategy

Literature on the diagnosis of AMA-negative PBC published from the period of Jan. 1950 to Mar. 2019 was searched in PubMed, MEDLINE, EMBASE, and the Cochrane Library. AMA-negative PBC with certain ANAs (including anti-gp210 and anti-sp100) was incorporated into the search strategy. The detailed search strategy was depicted in Supplementary 1: Table 1.

### 2.2. Inclusion and Exclusion Criteria

Inclusion criteria were as follows: (i) assessed the diagnostic accuracy of the ANA tests among AMA-negative PBC patients and controls; (ii) full-text articles; (iii) showed sufficient information of true positive (TP), false positive (FP), false negative (FN), and true negative (TN) numbers to calculate sensitivity and specificity; and (iv) the publication language should be in either English or Chinese.

Exclusion criteria were as follows: (i) review articles, case reports, and letters; (ii) lack of sufficient data; and (iii) articles without an abstract.

All the included studies were independently reviewed for eligibility by two investigators (Q.Z. and Z.L.). Disagreements on the inclusion of articles were resolved by consensus or involvement of an expert hepatologist with more than 10 years' experience in liver disease care and research (J.J.).

### 2.3. Diagnostic Criteria of PBC

The diagnosis of PBC can be established when two of the following three criteria are met: biochemical evidence of cholestasis based mainly on alkaline phosphatase elevation, presence of AMA, or histologic evidence of nonsuppurative destructive cholangitis affecting interlobular bile ducts [3].

### 2.4. Data Extraction

Data were retrieved from all the eligible studies independently by two investigators (Q.Z. and Z.L.). Studies with discrepancies in collection were referred to a senior methodologist (Y.K.) for resolution. The following variables were extracted: the first author, publication year, population, the control groups for diagnostic test, ANA type, and test results including TP, FP, FN, and TN numbers. The sensitivity and specificity for ANAs in the diagnosis of AMA-negative PBC were then calculated by reconstructing two-by-two tables.

### 2.5. Quality Assessment

The quality of the included studies was independently assessed by two reviewers with the Quality Assessment of Diagnostic Accuracy Studies-version 2 (QUADAS-2) scale [20]. This scale covered 4 domains in the assessment of risk bias (patient selection, index test, reference standard, and flow and timing). For each item, the answer should be provided as yes/no/unclear. “Yes” indicated a low risk of bias for this domain. “Unclear” presented a lack of details or uncertainty. “No” indicated a potential bias. Besides, applicability concerns were also assessed using these three domains including patient selection, index test, and reference standard. Low risk, unclear risk, and high risk were also clarified for the three domains of applicability. The disagreements would be settled by joint review with one senior methodologist (Y.K.).

### 2.6. Statistical Analysis

The sensitivities and specificities of ANA tests in AMA-negative PBC patients were pooled by diagnostic meta-analysis. The *Q* test and *I* ^2^ test were used to examine whether variations were caused by heterogeneity. The random-effects model was applied when the result of the *Q* test proved to be significant (*P* < 0.05 or *I* ^2^ > 50%) [21]. Subgroup analysis stratified by different types of ANAs and ethnicities was performed to evaluate the heterogeneities of sensitivities and specificities among subgroups for the diagnosis of AMA-negative PBC patients. The summary receiver operator characteristic (SROC) curve was calculated to evaluate the global performance. The areas under the SROC (AUSROC) curve represented the overall diagnostic accuracy of the ANA tests. Deeks' test was used to detect funnel plot asymmetry in reviews of diagnostic studies to investigate publication bias [22].

Statistical analysis was conducted with STATA 14.0 (StataCorp, College Station, TX, USA), Meta-DiSc 1.4 (XI Cochrane Colloquium, Barcelona, Spain), and Review Manager 5.3 (The Cochrane Collaboration, Oxford, UK). *P* value below 0.05 was considered statistically significant.

## 3. Results

### 3.1. Literature Search and Retrieval

**Figure 1:**
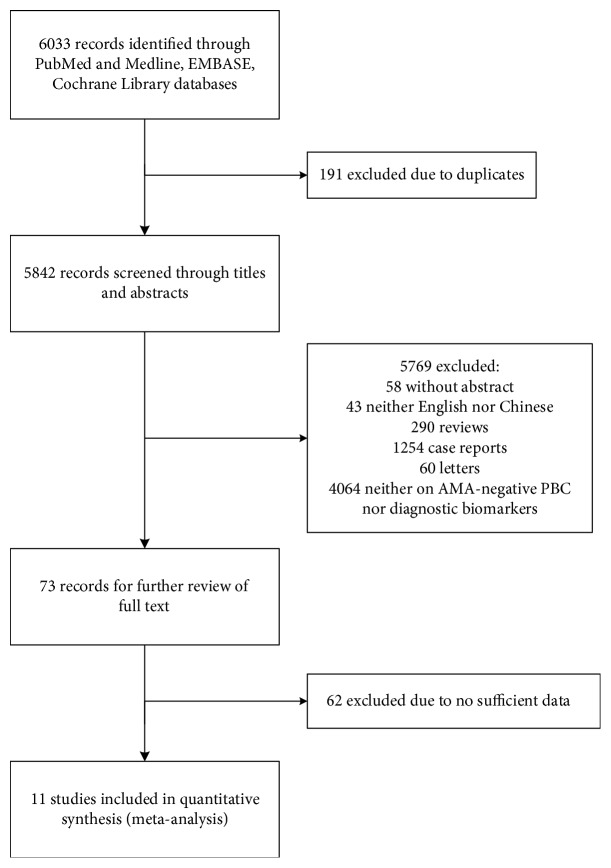
Flowchart of studies included in the meta-analysis. 5842 articles without duplicates were enrolled during database searching. Finally, 11 studies were included in quantitative synthesis and meta-analysis with the following inclusion criteria: (i) assessed the diagnostic accuracy of the ANA test on AMA-negative PBC patients and controls; (ii) full-text articles; (iii) showed sufficient information of true positive (TP), false positive (FP), false negative (FN), and true negative (TN) numbers to calculate sensitivity and specificity; and (iv) the publication language should be in either English or Chinese. The exclusion criteria were as follows: (i) review articles, case reports, and letters; (ii) lack of sufficient data; and (iii) articles without an abstract. Abbreviations: AMA: antimitochondrial antibody; PBC: primary biliary cholangitis.

The flowchart of the literature search process is illustrated in Figure 1. A total of 5842 articles without duplicates were identified through a predefined search strategy from PubMed, MEDLINE, EMBASE, and the Cochrane Library. The abstracts were screened, and 73 articles met the criteria for full-text review. Finally, 11 studies were included in the meta-analysis [16, 23–32].

### 3.2. Study Characteristics

A total of 11 studies with 400 AMA-negative PBC patients and 6217 control subjects were included for final analysis. There were 7 studies that tested both anti-sp100 and anti-gp210. Anti-sp100 was additionally tested in 1 study with PML and anti-sp140 [26]. Geographically, 3 studies were reported from Asia, 3 from North America, and 5 from Europe. Ethnically, 3 studies were conducted in Asian populations (2 from China and 1 from Japan) and 8 studies from Caucasians (5 from Italy, 2 from Canada, and 1 from America). The information of the control subjects and other characteristics including the publication year, country, controls, antibody types, and TP, FP, FN, and TN numbers are presented in Table 1.

### 3.3. Quality Assessment

**Figure 2:**
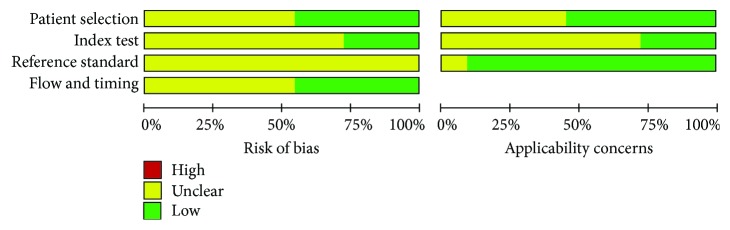
Quality assessment of diagnostic accuracy studies in the meta-analysis. Patient selection, index test, reference standard, and flow and timing were assessed of qualities. Patient selection, index test, and reference standard were considered in the applicability concerns. All the included studies were of moderate quality with yellow or greenbars. No high risk existed in these studies with no red bars. Abbreviations: High: high risk; Unclear: unclear risk; Low: low risk.

In the domain of patient selection, 5 studies (45.5%) had low risks of bias and another 6 studies (54.5%) had unclear risks of bias due to unclear description of consecutive patient selection. In the domain of index tests, 3 studies (27.3%) presented a blinded index test to reference standard and 8 studies (72.7%) indistinctly described whether or not the index tests were blinded to the reference standard. None of these studies presented a blinded reference standard to index test; as a result, the risks of bias were unclear in all the included studies. For the item of flow and timing, 5 studies (45.5%) described an appropriate interval between the index test and reference standard while the other 6 studies (54.5%) did not mention. The applicability concerns were the same with the risk of bias besides the reference standard. In these diagnostic studies, all the patients with AMA-negative PBC were selected with reference standard criteria; as a result, low bias occurred in the applicability concerns on the domain of reference standard (Figure 2).

### 3.4. Overall Sensitivity and Specificity of ANAs

**Figure 3:**
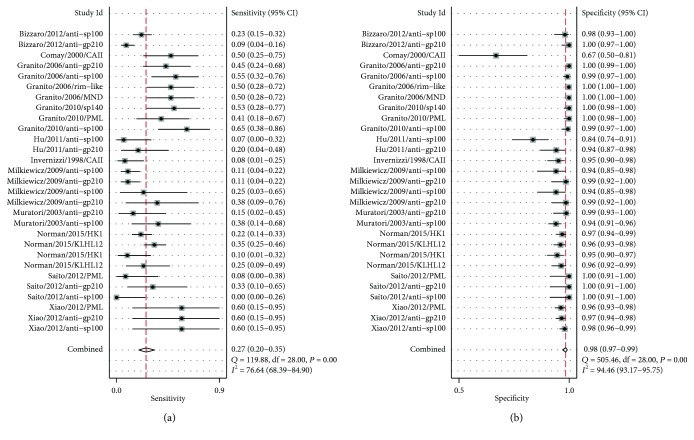
Forest plot of the sensitivity and specificity of ANAs for the diagnosis of AMA-negative PBC. In order to distinguish different ANAs, we listed both the author name with publication years and the different categories of ANAs. That will lead to one study with more than one forest plot in Figure 3. The first author, published years, and types of ANAs are shown together with sensitivities, specificities, and 95% confidence interval. Combined sensitivities and specificities are also shown with the results of the *Q* test and the *I* ^2^ test. Abbreviations: ANAs: antinuclear antibodies; AMA: antimitochondrial antibody; PBC: primary biliary cholangitis.

The reported sensitivities of the ANAs for diagnosis of AMA-negative PBC ranged from 0% to 65%, and the specificities ranged from 67% to 100%. Pooled analysis by random-effects models showed that the sensitivity and specificity of the ANAs were 27% (95% CI: 20%, 35%) and 98% (95% CI: 97%, 99%), respectively (Figure 3).

### 3.5. Sensitivity and Specificity for Anti-gp210 and Anti-sp100

**Figure 4:**
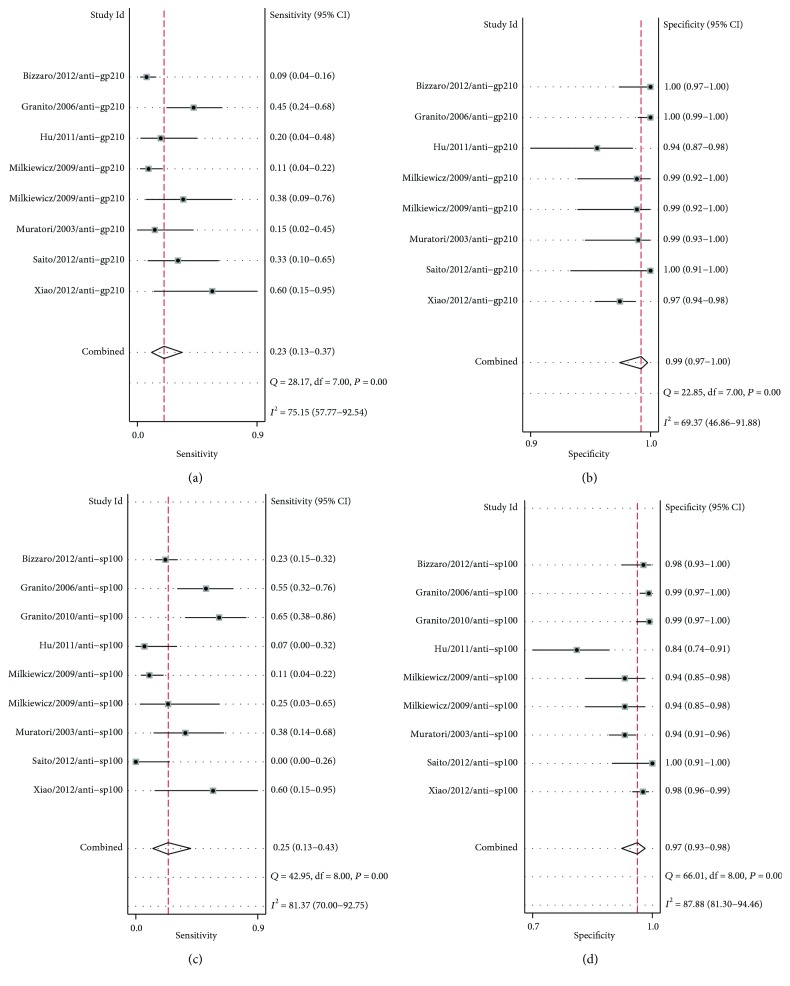
Forest plots of the sensitivity and specificity of anti-gp210 (a, b) and anti-sp100 (c, d) in the diagnosis of AMA-negative PBC. In order to distinguish different ANAs, we listed both the author name with publication years and the different categories of ANAs. That will lead to one study with more than one forest plot in the figure. The first authors and published years are shown together with sensitivities, specificities, and 95% confidence interval. Combined sensitivities and specificities are also shown with the results of the *Q* test and the *I* ^2^ test. Abbreviations: AMA: antimitochondrial antibody; PBC: primary biliary cholangitis.

**Figure 5:**
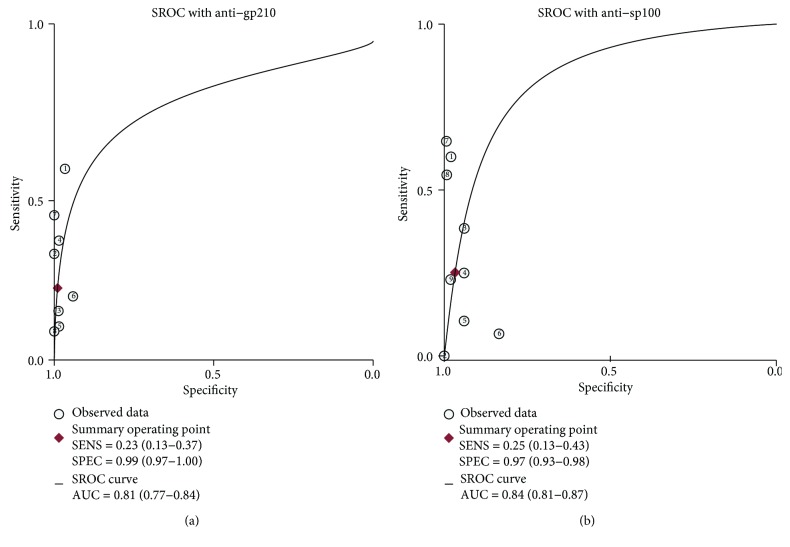
The SROC curve of anti-gp210 (a) and anti-sp100 (b) tests for the diagnosis of PBC. The summary receiver operator characteristic (SROC) curve was used for evaluating the global summary of test performance and the relationship between sensitivity and specificity. The area under the SROC (AUSROC) curve represented the overall performance of the detection method. Abbreviations: SROC: summary receiver operating characteristic; AUC: area under curves; PBC: primary biliary cholangitis.

Subgroup analysis stratified by the two main types of ANAs for diagnosis of AMA-negative PBC patients is presented here (Figure 4). For anti-gp210, the pooled sensitivity and specificity were 23% (95% CI: 13%, 37%) and 99% (95% CI: 97%, 100%), respectively (Figures 4(a) and 4(b)). For anti-sp100, the pooled sensitivity and specificity were 25% (95% CI: 13%, 43%) and 97% (95% CI: 93%, 98%), respectively (Figures 4(c) and 4(d)). The AUSROC curves for anti-gp210 and anti-sp100 were 0.81 (95% CI: 0.77, 0.84) and 0.84 (95% CI: 0.81, 0.87), respectively (Figure 5).

### 3.6. Subgroup Analysis by Ethnicity for Anti-gp210 and Anti-sp100

In the ethnicity subgroup analysis (depicted in Table 2), the results indicated that there were no significant differences of the pooled sensitivities and specificities in the various ethnicities among the total ANAs (23% vs. 28% and 97% vs. 99%). However, the sensitivities of anti-gp210 exhibited 31% (95% CI: 16%, 50%) in the Asian group and 18% (95% CI: 9%, 33%) in the Caucasian group. On the contrary, anti-sp100 appeared to possess a sensitivity of 20% (95% CI: 6%, 44%) in the Asian group and 30% (95% CI: 16%, 50%) in the Caucasian group (Table 2).

### 3.7. Analysis Compared with AMA-Positive PBC

To confirm whether the production of anti-gp210 and/or anti-sp100 antibodies is dependent on AMA-production or not, the sensitivities of anti-gp210 and anti-sp100 in AMA-positive PBC patients in the selected articles were also pooled for comparison (Supplementary 2: Table 2). The pooled sensitivity of anti-gp210 and anti-sp100 in AMA-positive PBC were 27% (21%, 36%) and 24% (19%, 29%), respectively. Results showed that anti-gp210 and anti-sp100 may be independent from AMA status.

### 3.8. Study Heterogeneity and Publication Bias

The results of the heterogeneity tests for overall sensitivity and specificity of ANAs were all significant (*P* < 0.01, *I* ^2^ = 76.64, and *P* < 0.01, *I* ^2^ = 94.46, respectively) (Table 2 and Figure 3). In the subgroup analysis divided by both ANAs and ethnicity, the pooled sensitivity and specificity for anti-gp210 in the Asian and Caucasian groups showed homogeneity in the subgroup analysis (*P* = 0.26 and 0.13 in the Asian group, *P* < 0.01 and *P* = 0.26 in the Caucasian group). However, heterogeneities of sensitivities and specificities among anti-sp100 for the diagnosis of AMA-negative PBC patients still existed even when considering ethnicity (Table 2).

**Figure 6:**
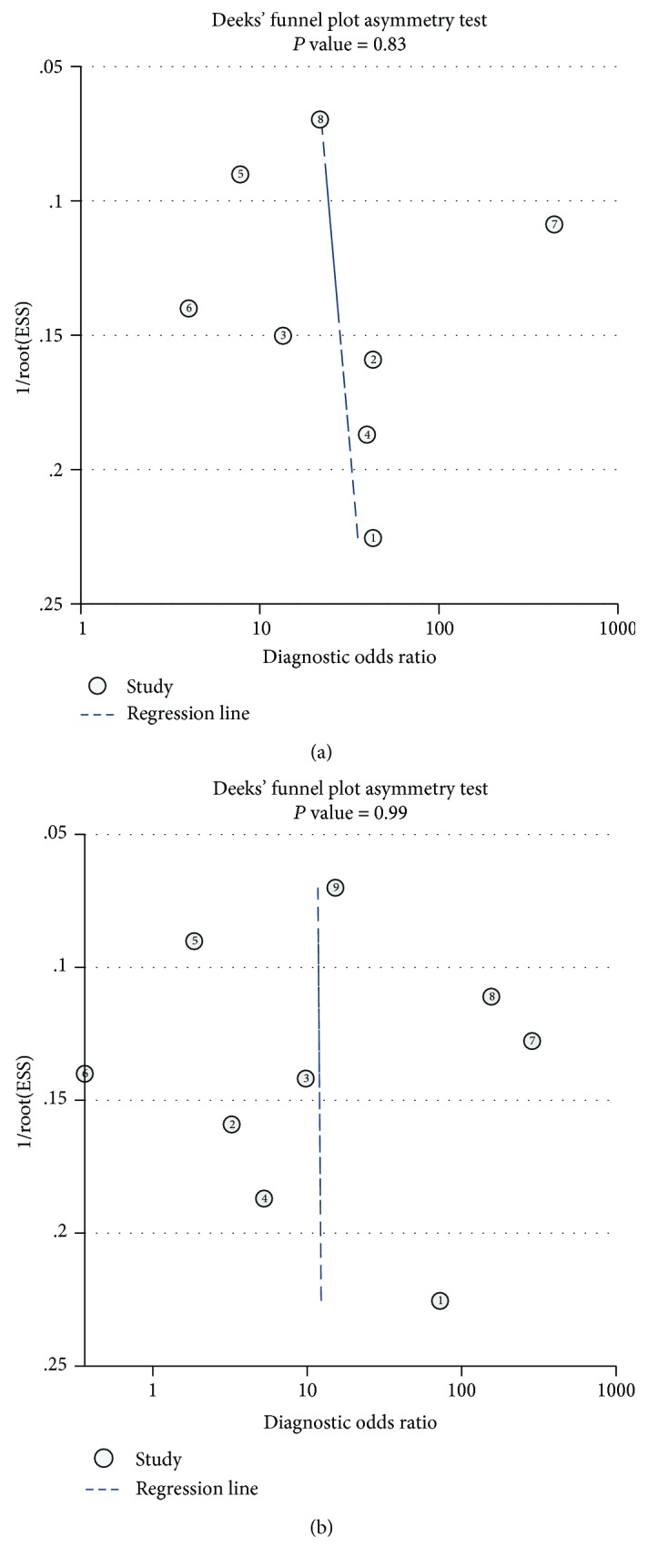
Funnel plot of included studies of anti-gp210 (a) and anti-sp100 (b). Deeks' test to detect funnel plot asymmetry in reviews of diagnostic studies was used to investigate publication bias. The almost vertical regression lines in the diagnostic odds ratios indicated there was no publication bias.

In Deeks' funnel plot asymmetry test, the *P* values of funnel plots for anti-gp210 and anti-sp100 were 0.83 (Figure 6(a)) and 0.99 (Figure 6(b)), respectively. The almost vertical regression lines in the diagnostic odds ratios indicated that no publication bias existed.

## 4. Discussion

In the current meta-analysis, we demonstrated that ANAs had high specificity and low sensitivity for diagnosis of AMA-negative PBC. Indeed, whereas the pooled specificities were over 95% for both anti-gp210 and anti-sp100, the pooled sensitivities were 23% and 25% for anti-gp210 and anti-sp100, respectively.

The current meta-analysis demonstrated that ANAs had a very high specificity for AMA-negative PBC. This finding aligns well with previous studies, which reported that the specificities of anti-gp210 and anti-sp100 for both AMA-positive and AMA-negative PBC patients were 97% and 99%, respectively [33, 34]. These results implied that anti-gp210 and/or anti-sp100 could be applied as a reliable rule-in biomarker for PBC. This is especially relevant for patients with high suspicion of PBC but negative for AMA and probably could reduce the necessity of liver histology in this setting [3]. Moreover, our study also demonstrated that the diagnostic performance of these two ANAs was similar in Asian and Caucasian populations.

In line with our findings, the overall positive rate of anti-gp210 or anti-sp100 was reported to be low in PBC patients, especially in AMA-negative PBC patients [34, 35]. In patients with AMA-positive PBC, the prevalence of anti-gp210 and anti-sp100 has been reported to be 16% to 18% and 24% to 31%, respectively [36].

Therefore, it is not surprising that the sensitivity of these two ANAs for diagnosis of AMA-negative PBC was rather low, which is also in line with a previous meta-analysis in that the sensitivities ranged from 26% to 29% for anti-gp210 and from 21% to 25% for anti-sp100 for the diagnosis of PBC patients (AMA positive or negative) [18]. It has been reported that a slightly better sensitivity could be achieved by combining the two biomarkers [32, 37]. All these results suggest that these two ANAs could not be used as reliable rule-out biomarkers for PBC.

Although the exact mechanism is unclear, similar pathogenic themes of liver injury have been postulated for AIH and PBC [38, 39]. Because their clinical and biochemical profiles have some overlap, these two diseases need to be differentiated from each other [40, 41]. Some studies have indicated that anti-gp210 and anti-sp100 were detected in 34% and 26% of PBC patients, whereas they were only seen in 7% and 16% of AIH patients [27]. However, Milkiewicz et al. reported that ANA-positive rates among patients with AMA-positive AIH or AMA-positive PBC were similar (60% vs. 59%) [29]. Therefore, further research is necessary to validate the diagnostic performance of anti-gp210 and anti-sp100 to differentiate AMA-negative PBC from AIH.

Several limitations exist in this meta-analysis. First, since AMA-negative PBC is a rare disease, the number of patients recruited by the original studies was usually not big and the ANA profiles were not homogeneous. Second, language bias may exist since studies published in non-English or non-Chinese language were not included in this meta-analysis. Fortunately, publications in other languages consisted of quite a low proportion (0.7%), which may not change the conclusion. Third, the PBC patients included in this meta-analysis mainly came from Italy and Canada and different control groups were used in the original studies; therefore, these may affect the external validity.

## 5. Conclusions

In conclusion, ANAs including anti-gp210 and anti-sp100 exhibited very high specificity but low sensitivity for the diagnosis of AMA-negative PBC, which therefore could be used as reliable biomarkers to reduce the necessity of liver histology.

## Figures and Tables

**Table 1:** Characteristics of the selected studies.

Author	Year	Country	AMA-negative PBC	Controls	Antibody type	TP (*N*)	FP (*N*)	FN (*N*)	TN (*N*)	Sensitivity (%)	Specificity (%)
Bizzaro et al. [23]	2012	Italy	100	104^a^	Anti-gp210	9	0	91	104	9.0	100.0
					Anti-sp100	23	2	77	102	23.0	98.1
Comay et al. [24]	2000	Canada	16	39^b^	CAII	8	13	8	26	50.0	66.7
Granito et al. [25]	2006	Italy	22	4248^c^	MND	11	5	11	4243	50.0	99.9
					Rim-like	11	6	11	4242	50.0	99.9
				262^d^	Anti-sp100	12	2	10	260	54.5	99.2
					Anti-gp210	10	0	12	262	45.5	100.0
Granito et al. [26]	2010	Italy	17	157^e^	Anti-sp100	11	1	6	156	64.7	99.4
					PML	7	0	10	157	41.2	100.0
					Anti-sp140	9	0	8	157	52.9	100.0
Hu et al. [27]	2011	China	15	85^f^	Anti-gp210	3	5	12	80	20.0	94.1
					Anti-sp100	1	14	14	71	6.7	83.5
Invernizzi et al. [28]	1998	Italy	26	142^g^	CAII	2	7	24	135	7.7	95.1
Milkiewicz et al. [29]	2009	Canada	8	67^h^	Anti-gp210	3	1	5	66	37.5	98.5
					Anti-sp100	2	4	6	63	25.0	94.0
			57		Anti-gp210	6	1	51	66	10.5	98.5
					Anti-sp100	6	4	51	63	10.5	94.0
Muratori et al. [16]	2003	Italy	13	283^i^	Anti-sp100	5	17	8	266	38.5	94.0
				75^j^	Anti-gp210	2	1	11	74	15.4	98.7
Norman et al. [30]	2015	America	20	165^k^	KLHL12	5	6	15	159	25.0	96.4
					HK1	2	9	18	156	10.0	94.5
			89	254^l^	KLHL12	31	10	58	244	34.8	96.1
					HK1	20	8	69	246	22.5	96.9
Saito et al. [31]	2012	Japan	12	40^m^	Anti-sp100	0	0	12	40	0	100.0
					Anti-gp210	4	0	8	40	33.3	100.0
					PML	1	0	11	40	8.3	100.0
Xiao et al. [32]	2012	China	5	296^n^	Anti-sp100	3	6	2	290	60.0	98.0
					Anti-gp210	3	10	2	286	60.0	96.6
					PML	3	11	2	285	60.0	96.3

Note: ^a^other chronic liver diseases including AIH-1, AIH-2, PSC, hepatitis B virus-related cirrhosis, hepatitis C virus-related cirrhosis, and AH; ^b^liver patients including AIH and ALD; ^c^non-PBC patients; ^d^HCV, AIH, PSC, SLE, RA, and SjS; ^e^AIH, PSC, and SLE; ^f^AIH and LDC; ^g^AIH, pSS, SSc, SLE, and healthy subjects; ^h^AIH, PSC, and undetermined cholangiopathy; ^i^AIH, PSC, HCV, SLE, pSS, RA, MCTD, and V; ^j^AIH, PSC, and SLE; ^k^PSC, ALF, SSc, and SLE; ^l^non-PBC patients, including PSC, AIH/PSC, AIH, SjS, UC, CD, HBV, HCV, HCC, VBDS, LS, and healthy donors; ^m^AIH; ^n^pSS, SLE, RA, AS, and SSc. The sensitivities and specificities among AMA-negative PBC patients in the selected studies are shown in this table, including the first author, publication year, country, number of antimitochondrial antibody- (AMA-) negative PBC, number of controls, number of true positive cases (TP), number of false positive cases (FP), number of false negative cases (FN), number of true negative cases (TN), sensitivity, and specificity. Abbreviations: AH: active hepatitis; AIH: autoimmune hepatitis; ALD: alcoholic liver injury; ALF: acute liver failure; AMA: antimitochondrial antibody; ANA: antinuclear antibodies; CAII: carbonic anhydrase II; CD: Crohn's disease; ELISA: enzyme-linked immunosorbent assay; FP: false positive; FN: false negative; HBV: hepatitis B virus; HCC: hepatocellular carcinoma; HCV: hepatitis C virus; HK1: hexokinase-1; IIF: indirect immunofluorescence; KLHL12: Kelch-like 12; LS: liver sarcoidosis; MCTD: mixed connective tissue disease; MND: multiple nuclear dot; PBC: primary biliary cholangitis; PML: promyelocytic leukemia protein; PSC: primary sclerosing cholangitis; pSS: primary Sjogren's syndrome; RA: rheumatoid arthritis; SLE: systemic lupus erythematosus; SSc: systemic sclerosis; TP: true positive; TN: true negative; UC: ulcerative colitis; V: vasculitis; VBDS: vanishing bile duct syndrome.

**Table 2:** Ethnicity subgroup analysis of ANAs in the diagnostic accuracy of ANAs.

Analyses	No. of studies	Pooled sensitivity (95% CI)	*I* ^2^	*P*	Pooled specificity (95% CI)	*I* ^2^	*P*	Pooled+LR (95% CI)	Pooled−LR (95% CI)	DOR (95% CI)
Total	11	0.27 (0.20, 0.35)	76.64	<0.01	0.98 (0.97, 0.99)	94.46	<0.01	17.1 (8.1, 36.4)	0.74 (0.67, 0.82)	23 (10, 53)
Asian	3	0.23 (0.10, 0.44)	64.68	0.01	0.97 (0.93, 0.99)	83.94	<0.01	6.9 (2.1, 23.2)	0.80 (0.63, 1.00)	9 (2, 35)
Caucasian	8	0.28 (0.21, 0.37)	79.01	<0.01	0.99 (0.97, 0.99)	95.90	<0.01	21.7 (8.6, 54.6)	0.72 (0.64, 0.82)	30 (11, 82)
Anti-gp210	7	0.23 (0.13, 0.37)	75.15	<0.01	0.99 (0.97, 1.00)	69.37	<0.01	21.9 (6.0, 80.4)	0.78 (0.67, 0.91)	28 (7, 111)
Anti-sp100	8	0.25 (0.13, 0.43)	81.37	<0.01	0.97 (0.93, 0.98)	87.88	<0.01	7.7 (2.3, 25.7)	0.77 (0.62, 0.96)	10 (2, 41)
Anti-gp210 in Asian^∗^	3	0.31 (0.16, 0.50)	26.80	0.26	0.96 (0.94, 0.98)	50.60	0.13	8.6 (3.6, 20.7)	0.70 (0.55, 0.89)	12 (4, 33)
Anti-gp210 in Caucasian	4	0.18 (0.09, 0.33)	81.03	<0.01	0.99 (0.98, 1.00)	24.55	0.26	32.4 (6.6, 157.9)	0.83 (0.71, 0.96)	39 (7, 213)
Anti-sp100 in Asian^∗^	3	0.20 (0.06, 0.44)	83.20	0.02	0.95 (0.92, 0.97)	95.50	<0.01	3.6 (0.01, 1491.4)	0.71 (0.14, 3.57)	5 (0, 1599)
Anti-sp100 in Caucasian	5	0.30 (0.16, 0.50)	82.90	<0.01	0.97 (0.94, 0.99)	77.72	<0.01	11.0 (3.1, 39.7)	0.72 (0.55, 0.93)	15 (3, 71)

The ethnicity subgroup analysis is shown in this table, including the subgroup, numbers of studies, pooled sensitivity, pooled specificity, pooled positive LR, pooled negative LR, and DOR. ^∗^The subgroup meta-analysis was performed in Meta-DiSc. Other subgroup meta-analyses were performed in STATA. Abbreviations: LR: likelihood ratio; DOR: diagnostic odds ratio.

## Data Availability

The extracted data used to support the findings of this study are included within the article. The search strategy data used to support the findings of this study are included within the supplementary information files. The diagnostic data including sensitivity and specificity for ANAs in the diagnosis of AMA-negative PBC data supporting this meta-analysis are from previously reported studies and datasets, which have been cited.

## Supplementary Materials

Supplementary 1 Table 1: the search strategy of this meta-analysis. The steps of the literature search on the diagnosis of antimitochondrial antibody- (AMA-) negative primary biliary cholangitis (PBC) published from Jan. 1950 to Mar. 2019 searched in PubMed, MEDLINE, EMBASE, and the Cochrane Library. The total search strategy included 29 steps. The first six steps limited the target disease to primary biliary cholangitis; the following steps from step 7 to step 28 limited the potential biomarkers to the diagnosis of PBC. Abbreviations: AMA: antimitochondrial antibody; AMA-M2: antimitochondrial antibody type 2; anti-M2: antimitochondrial antibody subtype m2; vcte: vibration-controlled transient elastography; GP-210 or GP210: nuclear pore membrane protein anti-gp210; PBC: primary biliary cholangitis; SP100 or SP-100: nuclear body protein anti-sp100.

Supplementary 2 Table 2: anti-gp210 and anti-sp100 antibodies in AMA-positive PBC patients. The sensitivities and specificities among AMA-positive PBC patients in the selected studies are shown in this table, including the number of antimitochondrial antibody- (AMA-) positive PBC, the number of controls, the number of true positive cases (TP), the number of false positive cases (FP), the number of false negative cases (FN), the number of true negative cases (TN), sensitivity, and specificity. Abbreviations: AH: active hepatitis; AIH: autoimmune hepatitis; ALD: alcoholic liver injury; ALF: acute liver failure; AMA: antimitochondrial antibody; ANA: antinuclear antibodies; CAII: carbonic anhydrase II; CD: Crohn's disease; ELISA: enzyme-linked immunosorbent assay; FP: false positive; FN: false negative; HBV: hepatitis B virus; HCC: hepatocellular carcinoma; HCV: hepatitis C virus; HK1: hexokinase-1; IIF: indirect immunofluorescence; KLHL12: kelch-like 12; LS: liver sarcoidosis; MCTD: mixed connective tissue disease; MND: multiple nuclear dot; PBC: primary biliary cholangitis; PML: promyelocytic leukemia protein; PSC: primary sclerosing cholangitis; pSS: primary Sjogren's syndrome; RA: rheumatoid arthritis; SLE: systemic lupus erythematosus; SSc: systemic sclerosis; TP: true positive; TN: true negative; UC: ulcerative colitis; V: vasculitis; VBDS: vanishing bile duct syndrome. Note: ^a^other chronic liver diseases including AIH-1, AIH-2, PSC, hepatitis B virus-related cirrhosis, hepatitis C virus-related cirrhosis, and AH; ^b^liver patients including AIH and ALD; ^c^non-PBC patients; ^d^HCV, AIH, PSC, SLE, RA, and SjS; ^e^AIH, PSC, and SLE; ^f^AIH and LDC; ^g^AIH, pSS, SSc, SLE, and healthy subjects; ^h^AIH, PSC, and undetermined cholangiopathy; ^i^AIH, PSC, HCV, SLE, pSS, RA, MCTD, and V; ^j^AIH, PSC, and SLE; ^k^PSC, ALF, SSc, and SLE; ^l^non-PBC patients, including PSC, AIH/PSC, AIH, SjS, UC, CD, HBV, HCV, HCC, VBDS, LS, and healthy donors; ^m^AIH; ^n^pSS, SLE, RA, AS, and SSc.
