# Supplementary material for: Meta-Analysis of Antinuclear Antibodies in the Diagnosis of Antimitochondrial Antibody-Negative Primary Biliary Cholangitis
Source: Gastroenterol Res Pract. 2019 Jun 10;2019:8959103. doi: 10.1155/2019/8959103 (PMC6590611; doi:10.1155/2019/8959103)
Supplement: Supplementary 1 — Table 1: the search strategy of this meta-analysis. The steps of the literature search on the diagnosis of antimitochondrial antibody- (AMA-) negative primary biliary cholangitis (PBC) published from Jan. 1950 to Mar. 2019 searched in PubMed, MEDLINE, EMBASE, and the Cochrane Library. The total search strategy included 29 steps. The first six steps limited the target disease to primary biliary cholangitis; the following steps from step 7 to step 28 limited the potential biomarkers to the diagnosis of PBC. Abbreviations: AMA: antimitochondrial antibody; AMA-M2: antimitochondrial antibody type 2; anti-M2: antimitochondrial antibody subtype m2; vcte: vibration-controlled transient elastography; GP-210 or GP210: nuclear pore membrane protein anti-gp210; PBC: primary biliary cholangitis; SP100 or SP-100: nuclear body protein anti-sp100. [file 8959103.f1.docx]

**Supplementary Materials**

**Supplementary Table1: The search strategy of this meta-analysis**

| Steps | Search strategies of the meta-analysis |
| --- | --- |
| #1. | "Liver Cirrhosis, Biliary"[Mesh] |
| #2. | "primary biliary cirrhosis" |
| #3. | "primary biliary cholangitis" |
| #4. | PBC |
| #5. | "humans"[Mesh] |
| #6. | #1 OR #2 OR #3 OR #4 AND #5 |
| #7. | anti-mitochondrial antibody |
| #8. | antimitochondrial antibody |
| #9. | AMA-M2 |
| #10. | anti-M2 |
| #11. | antimitochondrial antibody subtypes m2 |
| #12. | ama-negative |
| #13. | antimitochondrial antibody negative |
| #14. | anti-m2 negative |
| #15. | "Biopsy, Needle"[Mesh] |
| #16. | "Image-Guided Biopsy"[Mesh] |
| #17. | liver biopsy |
| #18. | Elasticity Imaging Technique* |
| #19. | Elasticity Imaging method* |
| #20. | vibration-controlled transient elastography |
| #21. | vibration controlled transient elastography |
| #22. | vcte |
| #23. | GP-210 |
| #24. | GP210 |
| #25. | SP100 |
| #26. | SP-100 |
| #27. | lamin b receptor |
| #28. | #7 OR #8 OR #9 OR #10 OR #11 OR #12 OR #13 OR #14 OR #15 OR #16 OR #17 OR #18 OR #19 OR #20 OR #21 OR #22 OR #23 OR #24 OR #25 OR #26 OR #27 AND #5 |
| #29. | #6 AND #28 |
