# Supplementary material for: Meta-Analysis of Antinuclear Antibodies in the Diagnosis of Antimitochondrial Antibody-Negative Primary Biliary Cholangitis
Source: Gastroenterol Res Pract. 2019 Jun 10;2019:8959103. doi: 10.1155/2019/8959103 (PMC6590611; doi:10.1155/2019/8959103)
Supplement: Supplementary 2 — Table 2: anti-gp210 and anti-sp100 antibodies in AMA-positive PBC patients. The sensitivities and specificities among AMA-positive PBC patients in the selected studies are shown in this table, including the number of antimitochondrial antibody- (AMA-) positive PBC, the number of controls, the number of true positive cases (TP), the number of false positive cases (FP), the number of false negative cases (FN), the number of true negative cases (TN), sensitivity, and specificity. Abbreviations: AH: active hepatitis; AIH: autoimmune hepatitis; ALD: alcoholic liver injury; ALF: acute liver failure; AMA: antimitochondrial antibody; ANA: antinuclear antibodies; CAII: carbonic anhydrase II; CD: Crohn's disease; ELISA: enzyme-linked immunosorbent assay; FP: false positive; FN: false negative; HBV: hepatitis B virus; HCC: hepatocellular carcinoma; HCV: hepatitis C virus; HK1: hexokinase-1; IIF: indirect immunofluorescence; KLHL12: kelch-like 12; LS: liver sarcoidosis; MCTD: mixed connective tissue disease; MND: multiple nuclear dot; PBC: primary biliary cholangitis; PML: promyelocytic leukemia protein; PSC: primary sclerosing cholangitis; pSS: primary Sjogren's syndrome; RA: rheumatoid arthritis; SLE: systemic lupus erythematosus; SSc: systemic sclerosis; TP: true positive; TN: true negative; UC: ulcerative colitis; V: vasculitis; VBDS: vanishing bile duct syndrome. Note: aother chronic liver diseases including AIH-1, AIH-2, PSC, hepatitis B virus-related cirrhosis, hepatitis C virus-related cirrhosis, and AH; bliver patients including AIH and ALD; cnon-PBC patients; dHCV, AIH, PSC, SLE, RA, and SjS; eAIH, PSC, and SLE; fAIH and LDC; gAIH, pSS, SSc, SLE, and healthy subjects; hAIH, PSC, and undetermined cholangiopathy; iAIH, PSC, HCV, SLE, pSS, RA, MCTD, and V; jAIH, PSC, and SLE; kPSC, ALF, SSc, and SLE; lnon-PBC patients, including PSC, AIH/PSC, AIH, SjS, UC, CD, HBV, HCV, HCC, VBDS, LS, and healthy donors; mAIH; npSS, SLE, RA, AS, and SSc. [file 8959103.f2.docx]

**Supplementary Table 2: Anti-gp210 and anti-sp100 antibodies in AMA-positive PBC patients.**

| **Author** | **AMA-positive PBC** | **Controls** | **Antibody type** | **TP**  **(N)** | **FP**  **(N)** | **FN**  **(N)** | **TN**  **(N)** | **Sensitivity**  **(%)** | **Specificity**  **(%)** |
| --- | --- | --- | --- | --- | --- | --- | --- | --- | --- |
| Bizzaro [23] | NA | 104^a^ | Anti-gp210 | NA | 0 | NA | 104 | NA | 100.0 |
|  |  |  | Anti-sp100 | NA | 2 | NA | 102 | NA | 98.1 |
| Comay [24] | 21 | 39^b^ | CA II | 9 | 13 | 12 | 26 | 42.9 | 66.7 |
| Granito  [25] | 101 | 4248^c^ | MND | 21 | 5 | 80 | 4243 | 20.8 | 99.9 |
|  |  |  | Rim-like | 12 | 6 | 89 | 4242 | 11.9 | 99.9 |
|  |  | 262^d^ | Anti-sp100 | 36 | 2 | 65 | 260 | 35.6 | 99.2 |
|  |  |  | Anti-gp210 | 24 | 0 | 77 | 262 | 23.8 | 100.0 |
| Granito [26] | 118 | 157^e^ | Anti-sp100 | 19 | 1 | 99 | 156 | 16.1 | 99.4 |
|  |  |  | PML | 14 | 0 | 104 | 157 | 11.9 | 100.0 |
|  |  |  | Anti-sp140 | 11 | 0 | 107 | 157 | 9.3 | 100.0 |
| Hu  [27] | 183 | 85^f^ | Anti-gp210 | 65 | 5 | 118 | 80 | 35.5 | 94.1 |
|  |  |  | Anti-sp100 | 50 | 14 | 133 | 71 | 27.3 | 83.5 |
| Invernizzi [28] | 189 | 142^g^ | CA II | 15 | 7 | 174 | 135 | 7.9 | 95.1 |
| Milkiewicz [29] | 30 | 67^h^ | Anti-gp210 | 11 | 1 | 19 | 66 | 36.7 | 98.5 |
|  |  |  | Anti-sp100 | 6 | 4 | 24 | 63 | 20.0 | 94.0 |
|  | 157 |  | Anti-gp210 | 26 | 1 | 131 | 66 | 16.6 | 98.5 |
|  |  |  | Anti-sp100 | 35 | 4 | 124 | 63 | 22.0 | 94.0 |
| Muratori [16] | 83 | 283^i^ | Anti-sp100 | 21 | 17 | 62 | 266 | 25.3 | 94.0 |
|  |  | 75^j^ | Anti-gp210 | 13 | 1 | 70 | 74 | 15.7 | 98.7 |
| Norman [30] | 80 | 165^k^ | KLHL12 | 11 | 6 | 69 | 159 | 13.8 | 96.4 |
|  |  |  | HK1 | 14 | 9 | 66 | 156 | 17.5 | 94.5 |
|  | 277 | 254^l^ | KLHL12 | 116 | 10 | 261 | 244 | 30.8 | 96.1 |
|  |  |  | HK1 | 146 | 8 | 131 | 246 | 52.7 | 96.9 |
| Saito  [31] | 52 | 40^m^ | Anti-sp100 | 8 | 0 | 44 | 40 | 15.4 | 100.0 |
|  |  |  | Anti-gp210 | 20 | 0 | 32 | 40 | 38.5 | 100.0 |
|  |  |  | PML | 5 | 0 | 47 | 40 | 9.6 | 100.0 |
| Xiao  [32] | 29 | 296^n^ | Anti-sp100 | 9 | 6 | 20 | 290 | 31.0 | 98.0 |
|  |  |  | Anti-gp210 | 12 | 10 | 17 | 286 | 41.4 | 96.6 |
|  |  |  | PML | 11 | 11 | 18 | 285 | 37.9 | 96.3 |
